# A Biologically Inspired Computational Model of Basal Ganglia in Action Selection

**DOI:** 10.1155/2015/187417

**Published:** 2015-11-10

**Authors:** Chiara Baston, Mauro Ursino

**Affiliations:** Department of Electrical, Electronic and Information Engineering “Guglielmo Marconi”, University of Bologna, Viale Risorgimento 2, 40136 Bologna, Italy

## Abstract

The basal ganglia (BG) are a subcortical structure implicated in action selection. The aim of this work is to present a new cognitive neuroscience model of the BG, which aspires to represent a parsimonious balance between simplicity and completeness. The model includes the 3 main pathways operating in the BG circuitry, that is, the direct (Go), indirect (NoGo), and hyperdirect pathways. The main original aspects, compared with previous models, are the use of a two-term Hebb rule to train synapses in the striatum, based exclusively on neuronal activity changes caused by dopamine peaks or dips, and the role of the cholinergic interneurons (affected by dopamine themselves) during learning. Some examples are displayed, concerning a few paradigmatic cases: action selection in basal conditions, action selection in the presence of a strong conflict (where the role of the hyperdirect pathway emerges), synapse changes induced by phasic dopamine, and learning new actions based on a previous history of rewards and punishments. Finally, some simulations show model working in conditions of altered dopamine levels, to illustrate pathological cases (dopamine depletion in parkinsonian subjects or dopamine hypermedication). Due to its parsimonious approach, the model may represent a straightforward tool to analyze BG functionality in behavioral experiments.

## 1. Introduction

The basal ganglia (BG) are a collection of subcortical structures, which are supposed to be implicated in many tasks, including action selection. While their role was traditionally restricted to motor function, more recent research focused on their involvement in cognition as well. Indeed, nowadays the implication of BG in a variety of cognitive functions has gained more and more consideration, as suggested by behavioral, clinical, and biochemical experiments in human and especially nonhuman beings [1–5]. These results are further supported by anatomical findings, demonstrating that the BG are connected to structures of the brain implicated in different cognitive tasks [1].

The large interest received by BG in recent neurophysiological and neuroscience research is motivated not only by their significant role in several motor and cognitive tasks in healthy state (action selection, categorization, working memory, etc…), but also by their malfunctions that lead to an array of diseases, primarily Parkinson disease (PD), a neurodegenerative disorder associated with a depletion of the dopaminergic neurotransmitter. Indeed, cognitive studies in humans, performed both in healthy controls and in PD subjects, documented cognitive deficits in PD patients, including deficits in memory, attention, learning, and solving visuospatial tasks [6–11]. The complexity of the mechanisms operating in the BG and the multiplicity of data acquired in recent years (biochemical, cellular, anatomical, functional, and behavioral) risk being insufficiently understood or poorly exploited if not incorporated into a coherent theoretical framework. Neurocomputational models provide an excellent way to summarize a large body of knowledge into a comprehensive setting. Indeed, the last 20 years have seen a growing literature investigating BG functions by means of computational modeling techniques (for a summary and a discussion of the most influential recent models, see the last section of this work, but also the excellent review papers by Cohen and Frank [12], Helie et al. [13], and Schroll and Hamker [14]). The goal of these models is to assess the mechanisms governing BG functions in rigorous quantitative terms; to this aim, they incorporate physiological knowledge on the different neural pathways implicated and on synaptic plasticity rules as well into a coherent structure, resulting in emerging properties and peculiar features that stand-alone field of sciences cannot explain yet. The final goal is to understand cognitive behavior and/or motor functions, in physiological as well as pathological conditions.

Despite the large number of valid models presented in the recent literature, there are still several aspects that deserve attention and may benefit from additional computer simulation studies.

This work presents a novel parsimonious model of BG in action selection, which combines rigor in the description of the main mechanisms and neural pathways with the simplicity of the general equations and is finalized at the interpretation of behavioral experiments. The work is motivated by the following main objectives (not all incorporated or simply insufficiently emphasized in previous studies):

(i) To introduce the role of cholinergic interneurons in BG mechanism and in synapse learning. Although various data underline that synapse learning in the striatum reflects a possible cooperation of dopamine and acetylcholine and may be mediated by cholinergic interneurons [15], we are aware just of a few models which consider this aspect explicitly, but assuming a different role for acetylcholine compared with our model [16, 17].

(ii) To analyze some important mechanisms operating in the BG. In particular, we will illustrate how a simple two-term Hebb mechanism for synapse learning can account for the capacity of BG to select new actions on the basis of a previous history of rewards and punishments. Moreover, we will provide clear examples on the role of the hyperdirect pathway during conflict resolution tasks. Although the latter 2 aspects have also been included in recent models [18], a thorough analysis of their functional role is still of value for the modeling community.

(iii) To show how the dopaminergic mechanism introduced in the model can account for various aspects of clinical relevance, such as the effect of dopamine depletion on the response times and on neglecting relevant responses.

## 2. Qualitative Model Description

### 2.1. Model Structure

**Figure 1:**
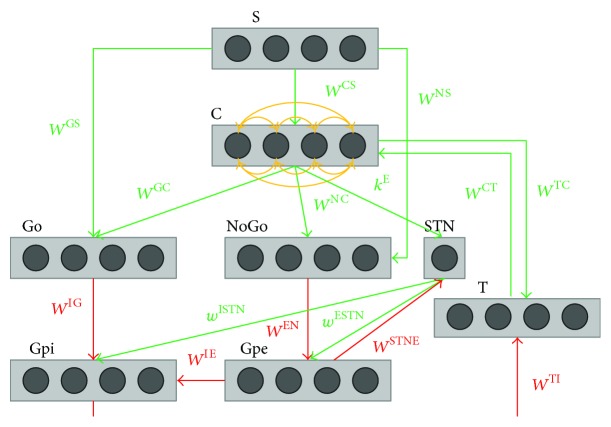
Graphical representation of the overall basal ganglia model. Rectangles represent different structures, circles neurons, arrows projections: green excitatory, red inhibitory, and orange lateral inhibition. This figure depicts the principal areas taken into consideration in the current model: the sensory representation of the stimulus in the cortex (S), the motor representation in the cortex (C), the thalamus (T), the striatum, functionally divided according to dopamine receptor expression (Go and NoGo), the subthalamic nucleus (STN), the globus pallidus pars externa (Gpe), and the globus pallidus pars interna (Gpi). The synapses where learning takes place are those from the cortex to the Go (*W* ^GC^) and the NoGo (*W* ^NC^) part of the striatum and those from the stimulus representation S to the Go (*W* ^GS^) and the NoGo (*W* ^NS^) part of the striatum.

The qualitative structure of the network is depicted in Figure 1.

The model includes a sensory representation in the cortex (S), the motor representation in the cortex (C), the thalamus (T), the striatum, functionally divided according to dopamine receptor expression (D1: Go, D2: NoGo), the subthalamic nucleus (STN), the globus pallidus pars externa (Gpe), and an output part represented by the globus pallidus pars interna (Gpi) and the substantia nigra pars reticulata (SNr) taken together. A peculiarity of the present model, compared to the majority of previous ones, consists in an explicit representation of the cholinergic interneurons (represented by the single unit ChI) and of their specific network.

The stimulus is represented in the cortex by a vector S. We assumed N different action channels, each one coding for a different alternative choice. These channels are segregated within the BG but interact within the motor prefrontal portion of the cortex via a winner-takes-all dynamics.

While all the other structures are modeled as N-neuron layers (representing N alternative segregated choices) the STN and ChI are modeled as single neurons since their activity represents a global property of the overall network; that is, they exert a global action on all channels.

In the following, each stimulus S in input is represented as a 4-element vector, and we also assume the presence of 4 segregated channels (i.e., we have 4 possible alternative choices, N = 4). We used just 4 action channels to reduce model complexity to a minimum, still allowing a thorough analysis of model dynamics: indeed 4 channels are sufficient to investigate the complexity of the relationships occurring when multiple possible choices are competing together.

Each neuron in the model is represented as a computational unit, which calculates its activity from the weighted sum of inputs. The output activity is in the range [0,1], representing a normalized firing rate of neurons. In particular, we used a sigmoidal static relationship to represent the presence of a lower threshold and upper saturation for neuronal activity and a first-order low-pass dynamics to mimic the integrative capacity of neuron membrane.

A first simplification with respect to biology is the use a single output region for the BG; that is, the globus pallidus pars interna and the substantia nigra pars reticulata are treated as a single region (named simply GPi hereafter). This is common to the majority of models of BG and is justified by their strict anatomical and functional similarities.

A further simplification consists in the use of dopamine (DA) directly as a modulating input factor, without explicitly representing the dopaminergic neurons in the substantia nigra pars compacta, which are responsible for the release of the dopaminergic neurotransmitter. This choice allows simple simulations of normal and pathological conditions, in which dopamine levels can be artificially altered by the disease or by external intervention.

The previous neurons are connected to realize the 3 main pathways (direct, indirect, and hyperdirect) which are known to work in the BG functioning.

As in the predominant “brake accelerator” view, the BG are only able to disinhibit a choice already selected by the cortex: in other words, the combined action of the 3 pathways only modulates the inhibition provided from the Gpi to the thalamus, thus consenting or blocking a response coded by the cortical neurons. Indeed, the thalamus receives only inhibitory projections from the BG, while the excitation is provided only by the selected neurons of the cortex C.

Let us follow the role and function of the main pathways in the model.

The input of the model is the vector S, which can be interpreted as a cortical representation of an external stimulus. S is connected both to the portion of the cortex C devoted to the implementation of the response (motor prefrontal) and to the striatum (both Go and NoGo), so that the striatum can contextualize the situation. We assume that a response is activated when the corresponding neuron in C overcomes a given “action threshold” (chosen close to 1: 0.95).

If there is no stimulus in input (i.e., the network is in its basal steady-state), the thalamus is globally inhibited since, without any excitation by the cortex, it receives only tonic inhibition by the Gpi [19]. On the contrary, an adequate stimulus can disinhibit the thalamus. In this sense, the model implements a winner-takes-all (WTA) mechanism, so that only the stronger response can be triggered. The WTA mechanism is attained through lateral inhibitions among the neurons in the cortex and a positive self-loop. The latter is realized by means of feedback connections between each neuron in the cortex and its corresponding representation in the thalamus (see Figure 1). The achievement of a sufficient activity by the winner requires the activation of this self-loop; hence that the corresponding representation in the thalamus can be previously disinhibited by the BG.

In order to regulate the thalamus inhibition/disinhibition, each neuron of the cortex is connected to its own Go (direct or striatonigral) and NoGo (indirect or striatopallidal) pathway via excitatory trained synapses, being the first pathway responsible for the focused facilitation of the response coded by the corresponding neuron and the second pathway responsible for its focused suppression.

Each striatonigral neuron in the Go pathway, in turn, sends an inhibitory synapse to the corresponding neuron of the Gpi: the more the neuron of the Go is excited, the more the neuron of the Gpi is inhibited, decreasing its tonic activity. Therefore, if the thalamus can be stimulated by the cortex, by means of this process the BG try to facilitate the gating of this specific response.

Similarly, each striatopallidal NoGo neuron sends an inhibitory synapse to the corresponding neuron of the Gpe, decreasing its tonic activity. This results in less inhibition provided to the Gpi, which thus becomes more active. By means of this complementary process, the BG try to stop the candidate action.

Indeed, it is the imbalance between the 2 pathways, due to different values of the synapses, that ultimately modulates the activity of the Gpi: if the Go pathway prevails, the Gpi provides less inhibition to the corresponding neuron of the thalamus (i.e., the BG “let go” the response); on the contrary if the NoGo pathway is more active, the Gpi provides more inhibition to the thalamus (i.e., the BG “stop” the response).

Each of Go and NoGo pathway runs in parallel for each neuron of the cortex [20] and this is how the network usually approaches the response selection task: a single neuron wins the competition in the cortex and a single action is selected. However, the choice for the cortex may become particularly difficult in the presence of a strong conflict among alternative candidates: in this case, the BG could provide fast but contradictory feedback and conflicting responses could win together (see the Results section). This challenging situations are managed by the hyperdirect pathway, carried out by the STN: its role is, indeed, to provide an overall stop signal to all the units of the Gpi in order to prevent any feedback by the BG and therefore let more time to the cortex to solve the conflict. In more detail, the STN receives an energy signal from the cortex, which summarizes the conflict level, and sends excitatory projections to all the neurons of the Gpi, providing an overall inhibition to the thalamus. Finally, in agreement with the physiological literature, we included a short loop between the Gpe and the STN (see Figure 1). Its role is mainly to control the STN activity, in order to avoid overactivity or undesired oscillations [21].

### 2.2. Dopamine and Acetylcholine

Evidences [2] show that basal ganglia are able to change their synaptic weights, in particular those between the cortex and the Go (*W* ^GC^) and the NoGo (*W* ^NC^) part of the striatum and similarly those between the stimulus representation S and the Go (*W* ^GS^) and the NoGo (*W* ^NS^) part of the striatum.

Moreover, dopamine is not uniquely excitatory or inhibitory but can exert different effects depending on the receptor: if it binds D1 receptor, it generally provides excitation, while if it binds D2 receptor it provides inhibition [20]. Hence, the effect of dopamine is different within the striatum, being primarily excitatory for the Go part and inhibitory for the NoGo part.

In more detail, a contrast enhancement phenomenon has been reported in the Go neurons [22]: if the activity of a Go neuron is high enough, dopamine produces further excitation, whereas it provides further inhibition to Go neurons with low activity. This results in a contrast enhancement effect. No similar effect has been reported so far for the NoGo neurons: in these neurons, dopamine exerts only an inhibitory effect.

In the model, dopamine exhibits a tonic level. Moreover, in case of punishment or reward, a phasic change in dopamine is produced (a transient dopamine peak during rewards; a transient dip during punishments). This induces a transient change in the activity of the neurons of the striatum, with the winning Go neurons generally receiving further excitation in case of reward, meanwhile the NoGo and losing Go neurons receive inhibition. On the contrary, in case of punishment, the Go neurons are inhibited, while all the winning NoGo neurons are excited.

A strong novelty introduced by the present model is the explicit description of the striatal cholinergic interneurons during learning, here represented by a single unit (ChI).

**Figure 2:**
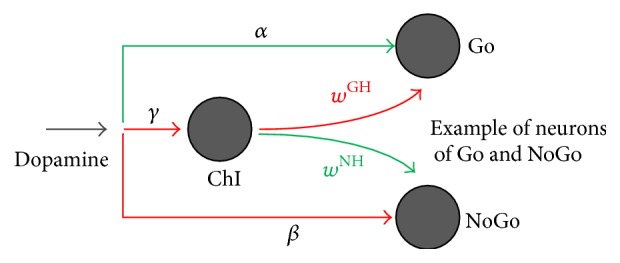
Focus on the effect of dopamine and cholinergic interneuron on Go and NoGo cells in the model. Arrows projections: green excitatory, red inhibitory.

The dependence of cholinergic interneurons on dopamine seems well-established in physiological literature: cholinergic interneurons express both D1 and D2 receptor [23, 24] and so, like the other striatal neurons, can sense dopamine changes. Furthermore, data in medical literature report a decrease in cholinergic activity following an increase in dopamine concentration, suggesting an inhibitory effect of dopamine on cholinergic interneurons [15]. Conversely, a fall in dopamine excites cholinergic neurons above their basal activity. Furthermore, we assumed that the activation of these interneurons provides inhibition to the Go neurons and excitation to the NoGo neurons, with an opposing role compared with dopamine. This role, despite being still debated in the literature, is in part supported by new research findings [25–28] (see also Discussion) but has never been assessed in previous neurocomputational models. A summary of the relationships between the cholinergic unit and the rest of the network is depicted in Figure 2.

Thank to this mechanisms, the cholinergic interneurons amplify the effect of dopamine on striatal neurons, especially during a dip in dopamine.

### 2.3. Hebb Rule and Learning

As discussed above, we assumed that a reward or punishment causes a phasic change in the dopamine level (a peak or a dip, resp.), which, in turn, is reflected in an opposite change in the cholinergic interneurons activity.

The phasic changes of the dopamine level and the consequent changes in the cholinergic interneurons activity affect the activity in the striatal neurons. This has an effect on synaptic plasticity.

Synaptic plasticity has been implemented in the model using a two-term Hebb rule, based on the correlation between presynaptic and postsynaptic activities, affecting all synapses entering into the striatal neurons (i.e., synapses *W* ^GC^, *W* ^NC^, *W* ^GS^, and *W* ^NS^ in the model). It is worth noting that this rule differs from the one adopted in most previous models, which generally use a three-term rule, including a multiplicative term directly modulated by phasic dopamine.

In the present model we did not use a temporal rule for training, but a simple associative rule based on the final value of neuronal activity. Both the steady-state presynaptic and the postsynaptic activities are compared with a threshold. Only if presynaptic activity is above threshold the synapse is eligible for training. In this way, just the synapses coming from active neurons in the cortex (the winners) and from significant stimuli in the sensory cortex can modify their value. The synapse is then reinforced (potentiation) or weakened (depression) depending on whether the postsynaptic activity is above or below threshold.

A fundamental aspect of our rule is that, in the absence of phasic dopamine changes, the activities of the winners (postsynaptic) striatal neurons are close to the threshold (we assumed a threshold as high as 0.5, denoting average activation). As a consequence, their synapses are not changed (or exhibit a minor change) since the postsynaptic activity term in the Hebb rule is close to 0. Conversely, a phasic dopamine change (a peak during reward or a dip during punishment), and the consequent phasic change in cholinergic activity, causes significant alterations in the activity of striatal neurons, moving them quite far from the postsynaptic threshold and inducing significant synaptic potentiation or depression.

In conclusion, transient changes in striatal activity due to dopamine and acetylcholine lead to activity driven plasticity, which is able to change network behavior and create new stimulus-response associations with experience, as rewards and punishment go on in time. As a consequence, synaptic plasticity induces a modification of the association between a specific stimulus S and the consequent response: previously rewarded outcomes will be more likely selected in the future, while punished ones will be actively avoided.

## 3. Mathematical Model Description

### 3.1. Individual Neuron Dynamics

In the following section, we will identify as *i* a postsynaptic neuron, receiving synapses *w*_*ij*_ from presynaptic neurons *j*, whose activity is *y*_*j*_. The neuron can eventually also have additional inputs coming from external sources not directly represented in the model: these are conveyed in a single term *I*_*i*_.

Synaptic and nonsynaptic inputs to the postsynaptic neuron *i* can be summarized in a synthetic variable *x*_*i*_. If there are N presynaptic neurons projecting to the postsynaptic neuron *i*, we can write

(1) $$\begin{array}{c} x_{i}=\overset{N}{\underset{j=1}{\sum }}w_{ij}y_{j}+I_{i}. \end{array}$$

In order to mimic the cell membrane integrative process, the input *x*_*i*_ is transformed in a postsynaptic variable *u*_*i*_, using a first-order differential equation with time constant *τ*:

(2) $$\begin{array}{c} \tau \frac{du_{i}}{dt}=-u_{i}+x_{i}. \end{array}$$

Finally, a sigmoidal function *ς* computes the activity of the neuron *i*, *y*_*i*_, from the output of the previous differential equation *u*_*i*_:

(3) $$\begin{array}{c} y_{i}=\varsigma \left(\;u_{i}\;\right). \end{array}$$

In the present model, the sigmoidal function *ς* was implemented as

(4) $$\begin{array}{c} y_{i}=\frac{1}{1+e^{-a\left(u_{i}-u_{0}\right)}}, \end{array}$$

where *a* and *u*_0_ are parameters which set the central slope and the central position of the sigmoid.

### 3.2. Network Connectivity

The spatial position of individual neurons is described by the subscripts *i*, *i* = 1,…, N, with N = 4 for the majority of the layers (S, C, Go, NoGo, Gpe, and Gpi). The STN and the cholinergic interneurons ChI, being both represented in the model as single units, do not need subscripts.

To describe connectivity in the network, synapses are represented with 2 superscripts and 2 subscripts. The 2 subscripts denote the position of the postsynaptic and presynaptic neurons, respectively. Superscripts denote the target layer (to which the postsynaptic neuron *i* belongs) and the donor layer (where the presynaptic neuron *j* is located), respectively. The acronyms used to indicate the individual layers are S: sensory cortex; C: motor cortex; T: thalamus; G: Go; N: NoGo; I: Gpi; E: Gpe; H: cholinergic interneurons ChI; STN: subthalamic nucleus. Moreover, *L* is used to denote the dynamics of lateral inhibition in the cortex.

To provide an example, the term *w*_*ij*_^GS^ denotes a synapse from the neuron at position *j* in the sensory cortex S, to a neuron at position *i* in the Go portion, G, of the striatum.

Referring to Figures 1 and 2, the nature of the synapses is represented by the specific color of the projections: excitatory projections are represented in green, while inhibitory ones are represented in red. Lateral inhibition is represented by orange arrows.

Among all the synaptic matrices and synaptic weights, we underline that a different denomination is used for *k* ^E^, since this projection does not connect single neurons but informs the STN about the conflict within the cortex C, expressed by means of an energy function *E*.

#### 3.2.1. Cortex

The first set of equations describe how the activity of the neurons in the cortex C is computed.

We can write for *i* = 1,…, N

(5) τLduiLdt=−uiL+∑j=1i≠jNlijyjC

(6) $$\begin{array}{c} \tau \frac{du_{i}^{C}}{dt}=-u_{i}^{C}+\overset{N}{\underset{j=1}{\sum }}w_{ij}^{CS}s_{j}+u_{i}^{L}+w_{ii}^{CT}y_{i}^{T} \end{array}$$

(7) $$\begin{array}{c} y_{i}^{C}=\varsigma \left(u_{i}^{C}\right). \end{array}$$

The previous equations can be explained as follows. Every neuron of the cortex C receives excitatory input from the whole stimulus S and an excitatory projection from the corresponding neuron in the thalamus. Moreover, it also receives an additional input *u*_*i*_^*L*^ reflecting lateral inhibition from the other neurons in the cortex. The latter is characterized by a different time constant *τ*_*L*_. If the neuron of the thalamus is active, the neuron of the cortex receives the positive feedback necessary to win the WTA selection.

#### 3.2.2. Go Part of the Striatum

The second set of equations describe the activity of the neurons in the Go.

We can write for *i* = 1,…, N

(8) τduiGdt=−uiG+∑j=1NwijGSsj+wiiGCyiC+α·DA·yiG−ϑG+wGHyH

(9) yiG=ςuiG.

As for the cortex C, every neuron of the Go receives excitatory input from the whole stimulus S and an excitatory projection from the corresponding neuron of the cortex C, starting here the direct pathway. In particular, it is worth noting that the matrix *W* ^GC^ is diagonal, reflecting the separation among the different action channels.

Dopamine (DA) and cholinergic interneuron activity (*y* ^H^) modulate the activity of each Go neuron.

Dopamine is excitatory (*α* > 0) if the Go activity is above a certain threshold (*ϑ*_G_), inhibitory on the contrary: this mechanism realizes the contrast enhancement effect [22].

The cholinergic interneurons are always inhibitory (*w* ^GH^ < 0) to the Go instead.

Both dopamine and acetylcholine exert tonic and phasic effects on Go activity.

#### 3.2.3. NoGo Part of the Striatum

The third set of equations describes the activity of the neurons in the NoGo.

We have for *i* = 1,…, N

(10) τduiNdt=−uiN+∑j=1NwijNSsj+wiiNCyiC+β·DA+wNHyH

(11) yiN=ςuiN.

Just like the Go, also every neuron of the NoGo receives excitatory input from the whole stimulus S and excitatory projection from the corresponding neuron in the cortex C, starting here the indirect pathway instead (hence, the matrix *W* ^NC^ is diagonal).

Dopamine (DA) and cholinergic interneuron (*y* ^H^) modulate the activity of each NoGo neuron as well, but in a different way.

Dopamine is always inhibitory (*β* < 0) to all the NoGo neurons, while the cholinergic interneurons provides excitation (*w* ^NH^ > 0) to the NoGo. Both dopamine and acetylcholine exert tonic and phasic effects on NoGo activity, in a specular way than in the previous Go case.

#### 3.2.4. Globus Pallidus Pars Externa

The fourth set of equations describe the activity of the neurons of the Gpe.

Equations are for *i* = 1,…, N

(12) τduiEdt=−uiE+wiiENyiN+wESTNySTN+IE

(13) yiE=ςuiE.

Every neuron of the Gpe receives an excitatory projection from the corresponding neuron of the NoGo part of the striatum, continuing the indirect pathway, while the excitation (*w* ^ESTN^) from the STN is part of a feedback loop to control STN activity, as previously mentioned.

Every neuron is tonically active at rest, thanks to the external input (*I* ^E^).

#### 3.2.5. Globus Pallidus Pars Interna

The fifth set of equations describe the activity of the neurons in the Gpi.

Equations are for *i* = 1,…, N

(14) τduiIdt=−uiI+wiiIGyiG+wiiIEyiE+wISTNySTN+II

(15) yiI=ςuiI.

Every neuron of the Gpi receives an excitatory projection from the corresponding neuron of the Go part of the striatum, continuing the direct pathway, and an inhibitory projection from the Gpe, while the excitation (*w* ^ISTN^) from the STN is part of the hyperdirect way. Indeed, the STN excites all the neurons of the Gpi, which in turns inhibit the corresponding neurons in the thalamus, thus braking any action selection.

Every neuron is tonically active at rest. In particular, the external input (*I* ^I^) overcomes the inhibitory input coming from the Gpe that is the reason why although the Gpe provides inhibition, the Gpi is active in the tonic state and inhibits the thalamus.

#### 3.2.6. Subthalamic Nucleus

The sixth set of equations describe the activity of the STN.

We can write (*y* ^STN^ and *u* ^STN^ are scalar variables)

(16) $$\begin{array}{c} \tau \frac{{du}^{\text{STN}}}{dt}=-u^{\text{STN}}+k^{E}E+\overset{N}{\underset{j=1}{\sum }}w_{j}^{\text{STNE}}y_{j}^{E} \end{array}$$

(17) with  E=∑i=1i≠jNyiCyjC

(18) $$\begin{array}{c} y^{\text{STN}}=\varsigma \left(u^{\text{STN}}\right). \end{array}$$

The STN is connected to the cortex C, but its activity does not depend on a single neuron, but on the overall activity of C, sensed by means of an energy function *E*. The latter reflects the conflict occurring in the cortex; that is, it signals the presence of 2 or more cortical neurons simultaneously highly active. The higher *E*, the higher the excitation of the STN. This is how the hyperdirect pathway starts.

The projection from the Gpe is part of the feedback loop to control STN activity, as previously said.

#### 3.2.7. Thalamus

The seventh set of equations describes the activity of the neurons in the thalamus T.

We have for *i* = 1,…, N

(19) τduiTdt=−uiT+wiiTIyiI+wiiTCyiC

(20) yiT=ςuiT.

Every neuron of the thalamus receives an excitatory projection from the corresponding neuron of the cortex C and an inhibitory projection from the corresponding neuron of the Gpi: the imbalance between the 2 determines whether the corresponding action is gated or not. It is worth noting that the excitation from the cortex to the thalamus realizes, together with the backward excitation from the thalamus to the cortex, a positive feedback loop, which is an essential part of the WTA cortical mechanism.

Every thalamic neuron is tonically silent at rest, as a consequence of the tonic activity at rest of the Gpi.

#### 3.2.8. Cholinergic Interneurons

The last set of equations describes the activity of the cholinergic interneurons ChI.

We have (*y* ^H^ and *u* ^H^ are scalar variables)

(21) τduHdt=−uH+IH+γ·DA

(22) yH=ςuH.

The cholinergic interneuron is inhibited (*γ* < 0) by dopamine (DA); hence it is influenced both tonically and phasically.

The ChI is tonically active at rest (*I* ^H^).

### 3.3. Synaptic Learning: The Hebb Rule

In this section we present the mathematical details of the Hebb rule specifically designed to reproduce synaptic plasticity as it occurs in our model of the BG.

Let Δ*w*_*ij*_^AB^ be the variation of the synapse between the presynaptic neuron *j* in layer B (B = S or C) and the postsynaptic neuron *i*, in layer A (A = G or N): the rule is expressed as follows:

(23) $$\begin{array}{c} \Delta w_{ij}^{AB}=\sigma {\left(\;y_{j}^{B}-\vartheta_{\text{PRE}}\;\right)}^{+}\left(\;y_{i}^{A}-\vartheta_{\text{POST}}\;\right), \end{array}$$

where the change in synaptic weight is due to the correlation between pre- and postsynaptic terms, comparing synaptic activities to specific thresholds.

The introduction of pre- and postsynaptic thresholds is a key element in the ability of this rule to reproduce plasticity due to the modulatory role of dopaminergic and cholinergic neurotransmitters.

The presynaptic term, with the use of the function “positive part” ([]+), detects where learning can actually occur: only the synapses coming from excited neurons of the cortex C or from salient stimuli in S, above the threshold *ϑ*_PRE_, can be modified. In particular, this means that only synapses from the chosen action (high value in C) and from the present context (high values in S) are subject to learning. The postsynaptic term considers whether the postsynaptic activity *y*_*i*_ (in our case in the striatal neuron) is above or below a certain threshold *ϑ*_POST_, which is set close to the tonic activity level of the winner without error feedback. It determines whether synaptic potentiation or depression occurs.

Finally, we assume that synapses cannot change their value (in particular all synapses entering the striatum are excitatory and cannot become negative) or cannot increase above a maximum saturation value. Hence, we have, for each trained synapse, 0 ≤ *w*_*ij*_^AB^ ≤ *w*_max⁡_ (with A = G or N and B = S or C).

### 3.4. Parameters Assignment

Since the model is extremely complex and contains many parameters, no automatic identification process was performed. Furthermore, most parameters describe average long-range connections among populations, for which neurophysiological data are not directly available. Hence, we used heuristic approach to tune the parameters.

In particular, parameters were tuned to respect a certain number of constraints, related first to the normal working point in the absence of external stimuli, then to the response to external stimuli, and finally to the effect of rewards and punishments.

- Individual neurons: the sigmoidal characteristics of individual neurons were given so that, in the absence of any input, the activity could be quite negligible (close to 0); the slope of the sigmoid allows a progressive increase from 0 to the upper saturation, thus consenting a fine modulation of neuronal activity. The time constant is the range normally adopted for rate neurons and agrees with the temporal dynamics resulting from more sophisticated integrate and fire models.
- Basal working point: in basal conditions the cortex, the thalamus, and the striatum must be inhibited; conversely the Gpi and Gpe exhibit a certain basal activity. We assumed that the basal activity of the Gpe is at about half the maximal activity; conversely, the basal activity of the Gpi is higher, close to the upper saturation. This high activity of the Gpi agrees with physiological data [19] and is necessary to maintain the thalamus inhibited. The previous constraints were realized by assigning values to the external inputs to the Gpi and Gpe and to the connectivity from Gpi to Gpe and from Gpe to the thalamus.
- Cortex and thalamus: the lateral connections within the cortex and the connections from the cortex to the thalamus and back from the thalamus to the cortex were assigned to realize quite a strong winner-takes-all mechanism. In particular, the cortico-thalamic loop represents a self-excitation, necessary to lead the winner neuron close to the upper saturation. The lateral inhibition is strong enough so that the winner neuron (close to saturation) can almost completely inhibit all the other cortical neurons. The synapses from the stimulus to the cortex have a moderate value so that, in the absence of thalamic excitation, a neuron in the cortex cannot reach a high activity level (hence the corresponding action is not triggered).
- Striatum: the synapses from the stimulus and from the cortex to the striatal (Go and NoGo) neurons were given moderate values before training, so that the striatal neurons in the active pathway (the winner) could have an intermediate activity between inhibition and upper saturation (approximately 0.5). This activity is close to the threshold of the Hebb rule. As a consequence, the corresponding synapses are reinforced or weakened only in response to reward or punishment feedbacks, which significantly alter the neuronal activity level. In the absence of feedback, the synapse changes are negligible.
- Globus pallidus: the synapses from the striatum to the Gpe and Gpi were given so that even a moderate activation of a striatal neuron (as a consequence of the cortical winner neuron and sensory inputs) could induce almost complete inhibition of the downstream neurons (in Gpi and Gpe). The synapses from Gpi to the thalamus ensure that when Gpi is active, the thalamus is completely inhibited. Hence, Gpi disinhibition corresponds to the desired gating mechanism.
- Subthalamic nucleus: the connectivity form the cortex to the STN was chosen so that even a moderate conflict (i.e., 2 cortical neurons simultaneously quite active) could excite the STN. The connection from STN to the Gpi ensures strong excitation of Gpi even at moderate activity of the STN, thus blocking any gating by the BG. Finally, the feedback connections between the STN and Gpe were chosen to allow a rapid deactivation of the STN when conflict is resolved.
- Dopamine and acetylcholine: parameters which set the dopamine action on striatal neurons were assigned so that a dopamine increase, during reward, could activate the winner Go neuron in the striatum close to its upper saturation and almost completely inhibit all NoGo neurons. Similarly, a dip in dopamine had to be able to strongly inhibit all Go neurons (also via activation of the cholinergic pathway) and excite the winner NoGo neuron. These constraints were satisfied by setting appropriate gains or synaptic weights from dopamine to striatum and from dopamine to cholinergic interneurons to striatum. As a consequence, the Hebb rule can work as requested, helping the Go pathway during reward and the NoGo pathway during punishment.

Starting from an initial cluster of values for the parameters, able to satisfy the constraints (i) and (ii), the technique was to assess the behavior of the network in order to have the desired behavioral output by progressively including subsequent constraints, fixing the previous parameters and determining the new ones.

Some parameters were obtained also including or knocking out specific structures, such as the STN.

This whole tuning procedure was iterated several times, considering cyclically the previous set of parameters and finally evaluating the entire neural network in order to verify if the whole behavior could satisfy all biological requirements.

The parameter values of the model in the default state and the initial values of state variables are reported in Table 1.

The synaptic weights of the model derived with the procedure described above are reported in Table 2 instead.

## 4. Results

In the following section, we describe some simulation results, to show how the present model works in paradigmatic conditions.

The majority of the simulations are run with a tonic dopamine value (0.45) corresponding, in our set of parameters, to healthy tonic levels. Simulations performed with a depletion or an increase in the dopamine level are clearly indicated.

### 4.1. Default Behavior

**Figure 3:**
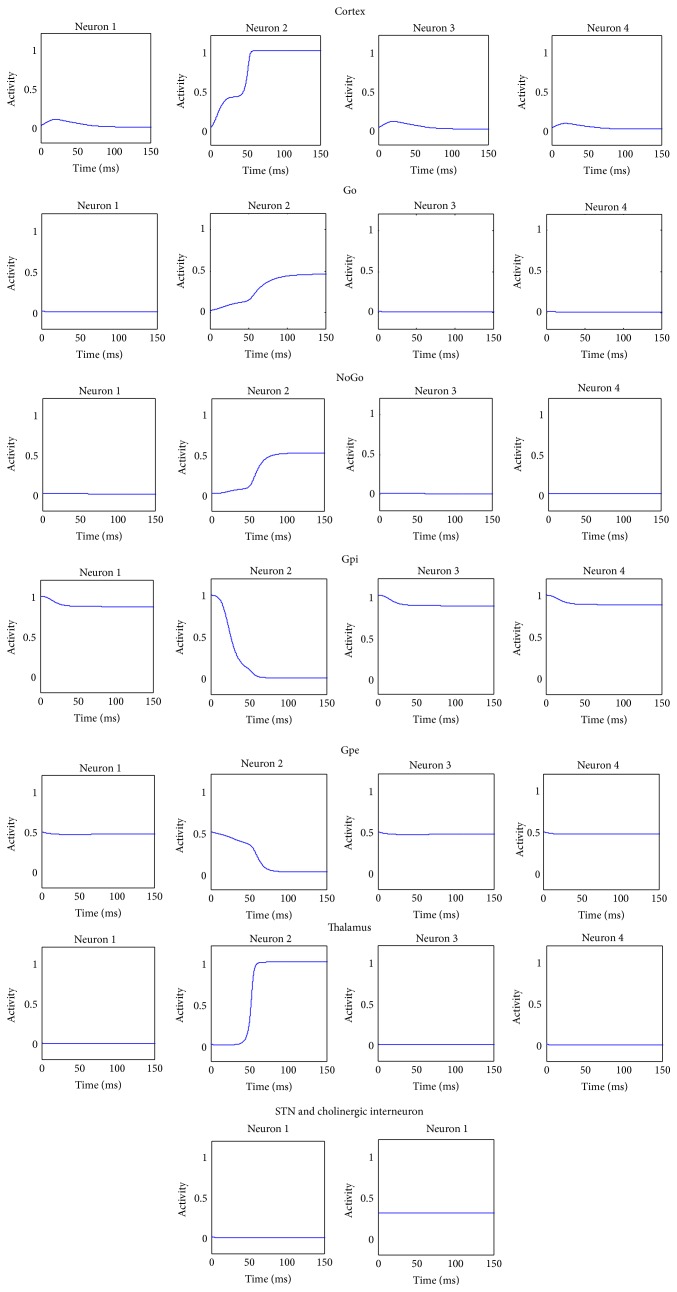
Temporal activity of the neurons in each structure of the whole network in a default case, assuming that the input stimulus favors the second response. In this case, the basal ganglia gate the response associated with the higher input stimulus (i.e., the second in this case): the second neuron in the cortex is maximally active, together with the corresponding neuron in the thalamus. The Go and NoGo pathways are both active, but the imbalance induces the correct response to be gated. In this case, the STN activity is low, indicating that the network does not perceive any conflict situation in the cortex.

An example of model simulation in the default case (i.e., with parameter values and the initial values of state variables as in Table 1 and synapses values as in Table 2, without learning) is presented in Figure 3. The stimulus given as input to the network was S = [0.3  0.8  0.3  0.2].

With the symmetrical basal values of synapses, the BG gate the response associated with the higher input stimulus (i.e., the second in this case): accordingly the second neuron in the cortex wins the competition and is maximally active, together with the corresponding neuron in the thalamus. The Go and NoGo portions show that both direct and indirect pathways are activated for the winning action. However, the correct response is gated due to an unbalance in the activity of the Go and NoGo neurons, resulting in a greater inhibition of the second neuron in the Gpi and therefore in less inhibition to the corresponding neuron of the thalamus. Hence, a positive loop occurs between the winning neuron of the cortex and the corresponding neuron in the thalamus.

The STN activity is low, signaling that the network does not perceive any conflict situation in the cortex.

Furthermore, the cholinergic interneuron activity is stable at its tonic level during the whole simulation, since no error feedback was released.

### 4.2. Conflict Resolution

**Figure 4:**
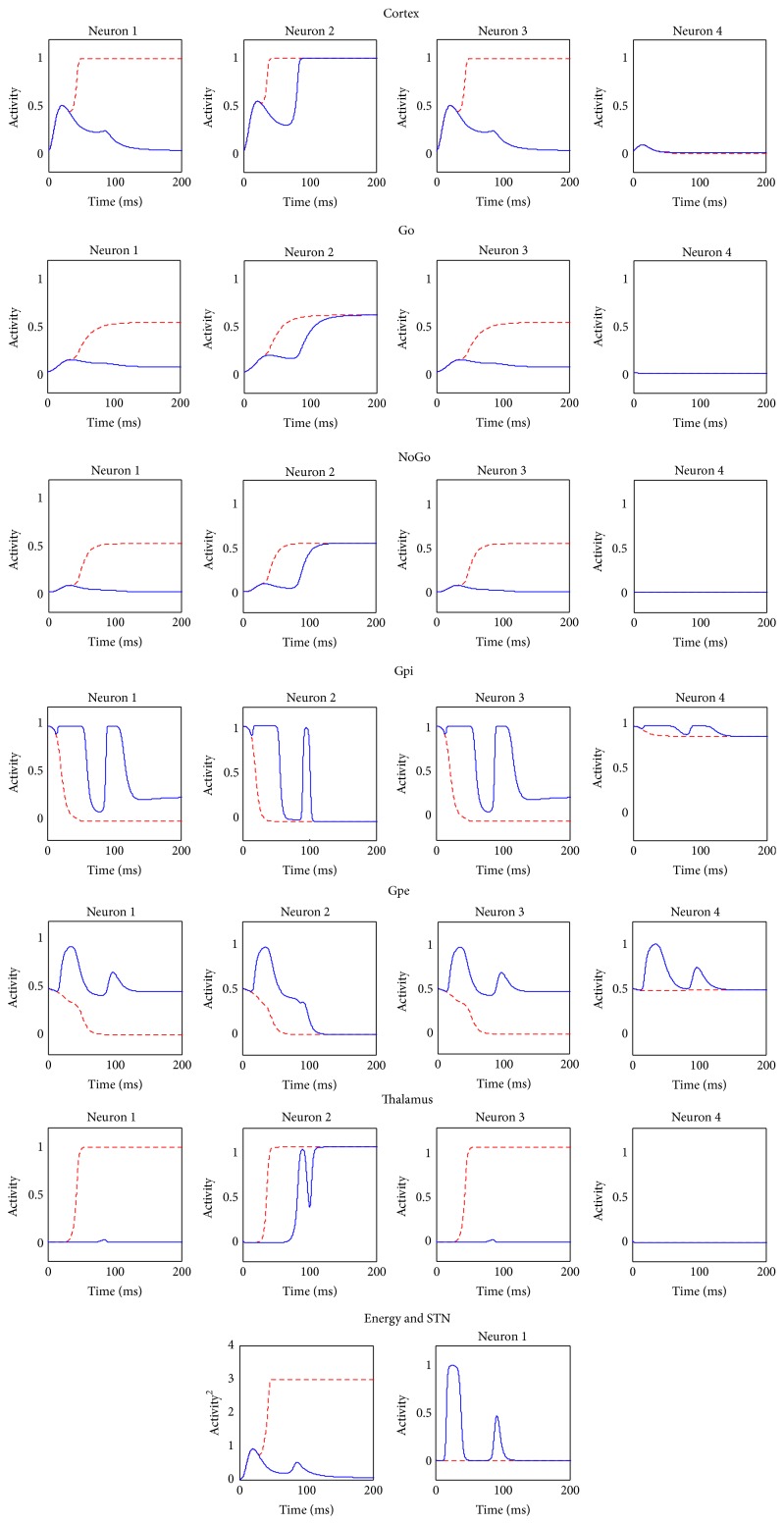
Temporal activity of the neurons in each structure of the whole network in a situation of strong conflict in the cortex, red dashed line with no STN, blue solid line with STN. The red signals of the cortex underline that 3 strong candidate actions in competition are quickly provided with basal ganglia feedback, being the STN off. All the corresponding 3 neurons of the thalamus are disinhibited, and the neurons of the cortex rapidly reach high levels of activation, so all the 3 are simultaneously gated. The blue signals underline that, in the presence of STN, just a single neuron of the cortex reaches the high value and is gated: the initial state of conflict is evident in the energy *E* function. The activity of the STN initially rises, temporarily stopping basal ganglia feedback until the conflict within the cortex is solved. As a consequence, the basal ganglia provide slower but correct feedback, allowing only 1 response to be gated.

In particular situations, when the choice for the cortex is particularly difficult, the hyperdirect pathway, carried out by the STN, works to prevent any gating by the BG. To analyze this situation, we used a conflicting stimulus S = [0.85  0.9  0.85  0.1] as input to the network. This stimulus creates a great conflict within the cortex, in particular among the first 3 neurons, although the second neuron receives the higher excitation. Results are illustrated in Figure 4.

The red dotted lines represent the results of a simulation performed by artificially eliminating the hyperdirect pathway (to this aim, the STN activity was set at 0 throughout the simulation). Here, a nonphysiological situation occurs, in which all the 3 candidate actions rapidly reach the high cortical activation. Both Go and NoGo signals for all the 3 actions rise, and the corresponding thalamic neurons are disinhibited. Of course, this situation is unacceptable, since 3 contradictory actions could be simultaneously gated.

The blue solid lines represent the same simulation performed assuming an intact STN: in this situation too an initial state of conflict is clearly evident looking at the cortical signals in C and at the energy function *E*. Consequently, the activity of the STN initially rises and temporarily stops basal ganglia feedback until the conflict within the cortex is solved. This is underlined also by the delay in the Gpi and in the thalamic activities, compared with the previous case.

The final result is that the cortex has more time to solve its conflict; as a consequence the BG provide correct feedback, even if slower: the final output response is the correct one, that is, that coded by the second neuron of C. More important, the model can select just 1 final response, avoiding conflicting experiences, despite the presence of multiple strong inputs.

Once the role of STN is accomplished, its activity is less necessary and essentially the neuron becomes silent.

### 4.3. Reward and Punishment

In a previous section we mentioned that dopamine and cholinergic interneurons phasic changes are responsible for synaptic plasticity in the BG. Here we present how error feedbacks (i.e., reward and punishment) are able to alter striatal activity, which is at the basis of synaptic plasticity described by our Hebb rule.

**Figure 5:**
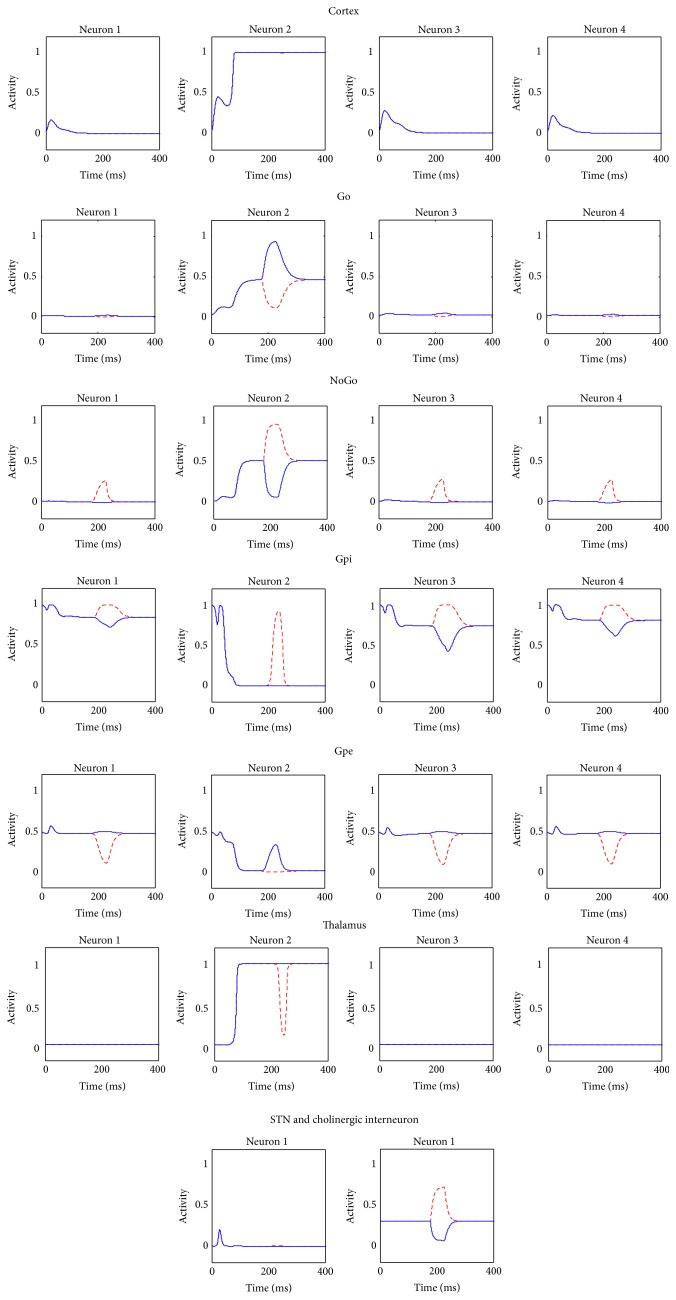
Temporal activity of the neurons in each structure of the whole network during error feedback, red dashed line punishment, blue solid line reward. The effect of reward and punishment is clearly noticeable in the activity of striatal neurons. In case of punishment (red signals), a transient dip occurs in the activity of the winning Go neuron. Conversely, the NoGo units clearly show a peak, particularly pronounced in the unit of the winning neuron. Furthermore, we can notice a transient peak in the cholinergic interneuron activity, underlying the inhibitory role of dopamine on ChI. In case of reward (blue signals), a transient peak occurs in the activity of the winning Go unit. The others Go units maintain low activities, due to the contrast enhancement effect of dopamine. All the NoGo neurons exhibit a transient dip in activity, particularly remarkable for the unit in the selected action channel. Finally, the activity of the cholinergic interneuron exhibits a transient dip, which contributes to the excitation of the Go pathway and to the inhibition of the NoGo pathway.

In the following simulations (Figure 5) we illustrate the effect that a reward or a punishment can have on the different activities in the network. The stimulus used is S = [0.4  0.8  0.6  0.5]. In the default state (i.e., without training) the gated response is the one coded by the second neuron of C, since it receives the greater excitation.

In the first simulation (red dotted lines) we assumed that the final response of the network receives a punishment. This is simulated by decreasing the dopamine level from its basal value (0.45) to zero. The dopamine dip starts at *t* = 100 ms, when the network has reached its steady-state level, and lasts for 50 ms (values suitable for latency and duration of phasic dopaminergic response [2]). In the second simulations (blue solid lines) we assumed that a reward occurs, simulated with a peak of dopamine (from 0.45 to a value double the normal, i.e., 0.9) still between 100 and 150 ms.

The effect of reward and punishment does not appreciably alter the activity in the cortex, but it is clearly noticeable in the activity of striatal neurons. In case of punishment, a transient dip occurs in the activity of the winning Go neuron. Conversely, the NoGo units clearly show a peak, particularly pronounced in the unit of the winning neuron. Furthermore, we can notice a transient peak in the cholinergic interneuron activity, underlying the inhibitory role of dopamine on ChI.

In case of reward, a transient peak occurs in the activity of the winning Go unit. The others Go units maintain low activities, due to the contrast enhancement effect of dopamine. All the NoGo neurons exhibit a transient dip in activity, particularly remarkable for the unit in the selected action channel. Finally, the activity of the cholinergic interneuron exhibits a transient dip, which contributes to the excitation of the Go pathway and to the inhibition of the NoGo pathway.

In conclusion, as a consequence of the dopamine transient changes, the phasic activity in the striatum, both in case of reward and punishment, is moved far from the postsynaptic threshold, thus allowing a significant Hebb learning of the incoming synapses.

### 4.4. Contribution of the Cholinergic Interneurons to Rewards and Punishment

**Figure 6:**
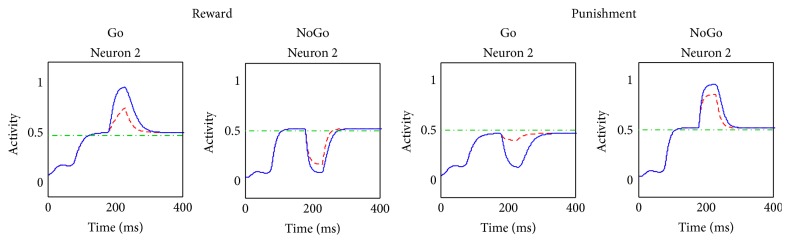
Temporal activity in the winner action channel of Go and NoGo neurons during reward and punishment, in the same conditions as in Figure 5: red dashed line with no phasic activity of ChI (the activity of the ChI was artificially constrained at a constant basal level), blue solid line with phasic activity of ChI. The red dotted signals have lower peaks and higher dips; this means that the contribution of ChI is essential to move the striatal activities far from the established threshold *ϑ*_POST_ (this threshold is shown with a green dot-and-dashed line) thus allowing greater changes in synaptic weights according to our specific Hebb rule.

One of the main novelties of the present model is the introduction of cholinergic interneurons and the simulation of their role in synaptic plasticity. In order to clarify the function of this specific mechanism, the previous simulations were repeated by artificially maintaining the activity of the ChI at a constant basal level. A comparison between the normal condition (blue solid line) and the absence of ChI (red dotted line) is displayed in Figure 6.

Since, during reward and punishment, the main changes in activity occurred in the striatal neurons (both Go and NoGo) in the selected action channel (i.e., the second channel in Figure 5), only the activity of these 2 neurons is displayed again in Figure 6.

The red dotted signals have lower peaks and higher dips: this means that the contribution of ChI is essential to move the striatal activities far from the established threshold *ϑ*_POST_ (this threshold is shown with a green dot-and-dashed line) thus allowing greater changes in synaptic weights according to our specific Hebb rule.

In particular, it can be noticed that the contribution of ChI is particularly essential for the Go neurons, especially during punishment; without this mechanism, the postsynaptic term in the Hebb rule would be close to 0, preventing any plasticity along the direct pathway.

### 4.5. Training

Basal ganglia can change their behavior and their stimulus-response associations by means of synaptic plasticity.

Given a stimulus S, the aim of training is to shift the chosen response from the default, prepotent one (in the previous simulations the one associated with the strongest element of S) to the one coded by another cortical neuron.

To train the network, we assumed a stimulus S = [0.15  0.15  0.9  0.7]. In the default state, the third cortical neuron receives the stronger excitation and therefore the third action is selected. Our aim is to progressively suppress the response coded by the third neuron of the cortex, when this stimulus S is presented in input to the network, and to train the network to gate the desired response, that is, the one coded by the fourth neuron.

In these simulations some noise (normal distribution with mean value 0 and standard deviation 0.25) was added to the original stimulus S. Since each element of the stimulus S has to be in a precise range of values previously specified, after noise addition each element of S was checked and eventually compelled in the range [0,1]. The addition of noise allows the gating of alternative actions in response to the same external input, and the occurrence of both reward and punishment during the different epochs. This means that the subject is not only exploiting the previous knowledge (coded in the synapse values) but also exploring new possibilities. This exploration/exploitation tradeoff is essential for having an efficient learning.

The training consisted of 100 epochs.

**Figure 7:**
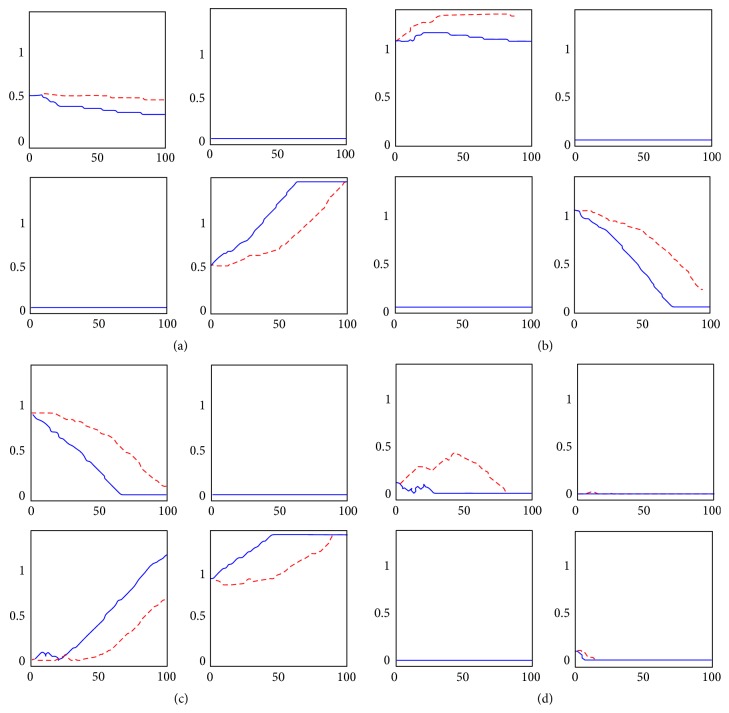
Synapses update during the 100 epochs of training, red dashed line with no phasic activity of ChI, blue solid line with phasic activity of ChI. In these simulations (contrarily to Figure 3) we assumed that, before training, the input stimulus favors the third response. However, during training, the third response is punished and the fourth is rewarded. Each panel represents the submatrix *W* ^IJ^ (3: 4; 3: 4). Row *i* is post-synaptic neuron *i*, while column *j* is presynaptic neuron *j*. (a) *W* ^GC^  (3: 4; 3: 4). (b) *W* ^NC^  (3: 4; 3: 4). (c) *W* ^GS^  (3: 4; 3: 4). (d) *W* ^NS^  (3: 4; 3: 4). Recalling that *W* ^GC^ is implied in the direct pathway, the decrease of the element in the position (3,3) disfacilitates the prepotent response. Conversely, the increase of the element in the position (4,4) facilitates the desired response. Recalling that *W* ^NC^ is implied in the indirect pathway, the increase of the element in the position (3,3) helps suppress the prepotent response. On the contrary, the decrease of the element in the position (4,4) provides less inhibition to the desired response. Similar consideration can be made for *W* ^GS^ and less intuitively for *W* ^NS^. It is evident from the red signals that the effect of the lack of acetylcholine induces a generally slower learning process, with synapses changing less compared to the normal case.

The changes in synapses *W* ^GC^, *W* ^NC^, *W* ^GS^, and *W* ^NS^, during the various epochs are illustrated in Figure 7 (blue solid lines). Since the important synaptic changes occurred only in the third and fourth action channels (where the presynaptic activity is high) just the submatrices involved in the third and fourth action channels, that is, *W* ^GC^  (3: 4; 3: 4), *W* ^NC^  (3: 4; 3: 4), *W* ^GS^  (3: 4; 3: 4), and *W* ^NS^  (3: 4; 3: 4), are portrayed for briefness.

(i) Synapses from cortex to Go: recalling that *W* ^GC^ is implied in the direct pathway, the decrease of the element in the position (3,3) disfacilitates the prepotent response. Conversely, the increase of the element in the position (4,4) corresponds to an increase in the facilitation of the desired response, with the corresponding synapse increased to its upper saturation.
(ii) Synapses form cortex to NoGo: recalling that *W* ^NC^ is implied in the indirect pathway, the slight increase of the element in the position (3,3) tends to suppress the prepotent response. On the contrary, the decrease of the element in the position (4,4) provides less inhibition to the neuron coding for the desired response; in this case the corresponding synapse is decreased to 0 (lower saturation).
(iii) Synapses from the sensory cortex to the Go: recalling that *W* ^GS^ is implied in the direct pathway, the decrease of the element in the position (3,3) and the lack of increase of the element in the position (3,4) provide less facilitation to the prepotent response. Similar to *W* ^GC^, this time 2 synapses, (4,3) and (4,4), rise to provide more facilitation to the desired response.
(iv) Synapses from the sensory cortex to the NoGo: *W* ^NS^ is implied in the indirect pathway, and its changes are less immediate to understand. Indeed, this matrix exhibits only mild variations as a consequence of training. Some changes (suppression of the third action by an increase in element (3,3)) are evident only during the first epochs and therefore contribute significantly just to the first phase of training. The reason is that, during the last epochs, punishments occur only rarely (since the network learned the correct strategy) and rewards are predominant, which inhibits all NoGo neurons, while the same elements of the input stimulus S are still high. Hence, because of the Hebb rule, all synapses from the high values of S to the NoGo are progressively decreased. This aspect might be improved by reducing the dopamine peaks in case of expected rewards (see Discussion).

**Figure 8:**
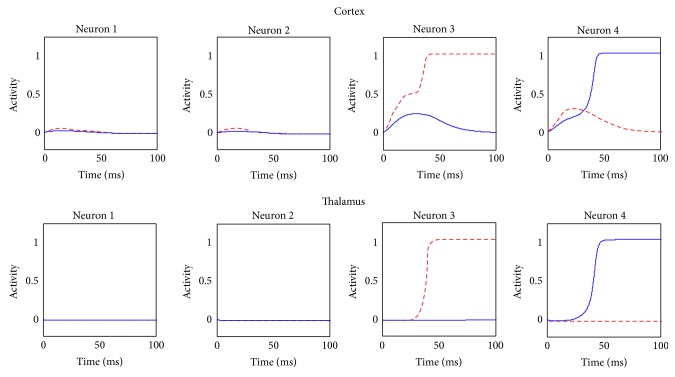
Comparison of model decision before and after training (with intact ChI). Temporal activity of the neurons in the cortex and in the thalamus: red dashed line before training, blue solid line after training. It is evident that synaptic plasticity is able to introduce a different stimulus-response association.

However, despite this incongruence, the comparison between the cortical and thalamic activities at the beginning and at the end of the training (Figure 8), in response to the same stimulus S, shows that the effect of synapses learning has been successful and that the network after the training is now able to adapt the stimulus-response coded as desired. This figure shows the temporal response of the network to the stimulus S = [0.15  0.15  0.9  0.7], given without noise, in the initial stage (red dotted signals) and in the final stage (blue solid signals) of training.

Before training, the prepotent response is gated, as shown by the final activity both in the cortex and the thalamus. The first weak sign of training is shown by a little dip in the activity of the third neuron of the thalamus, showing that the training is starting punishing the prepotent response, as expected. After 100 epochs of training, the network presented the same stimulus S but now the BG gate the desired response, showing that the training process was successful.

Finally, we repeated the training procedure by lesioning the ChI, as was done in the simulation of Figure 6 (the activity of the ChI is artificially maintained at a constant basal level, suppressing any phasic activity). The results are shown in Figure 7 with red dashed lines. It is evident that the effect of lower peaks and higher dips in Go/NoGo neurons, due to the lack of acetylcholine (previously shown in Figure 6), induces a slower learning process, with synapses changing less compared with the normal case, despite the same epochs of training. Therefore, cholinergic interneurons are proven to be essential to perform correct synaptic plasticity.

### 4.6. Network with Low, Normal, and High Tonic Dopamine Levels

In our model dopamine is present both in its tonic and in its phasic form.

The implication of phasic dopamine in synaptic plasticity was widely discussed before. Now we wish to focus on how the model is able to reproduce behavioral changes due to different levels of tonic dopamine and how tonic dopamine affects BG behavior in our model.

**Figure 9:**
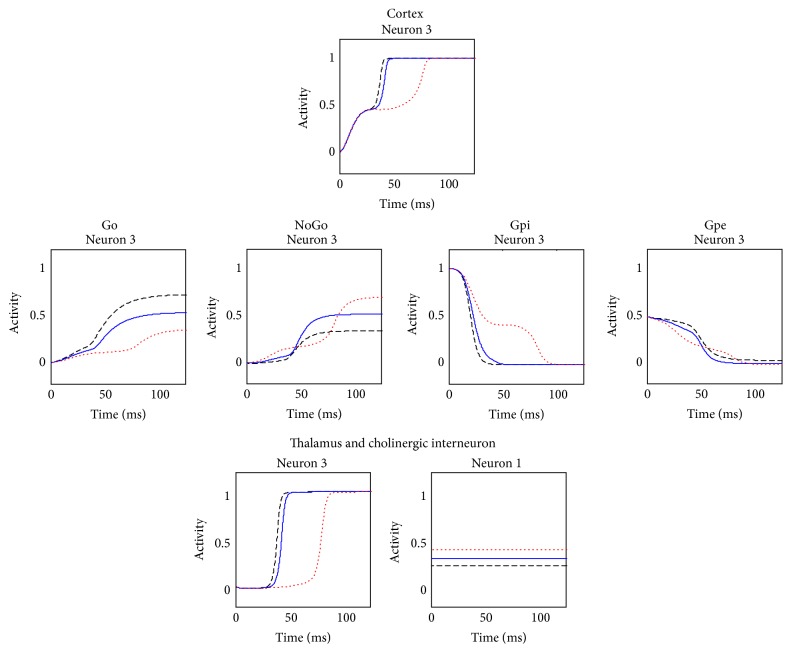
Temporal activity of the winning action channel in each structure of the whole network with different tonic dopamine levels, red dotted line low level, blue solid line normal level and black dashed line high level. No training was performed before. The tonic level of dopamine has effects on each structure of the network. In the cortex C, the higher the tonic dopamine level, the faster the response, caused by a prompter feedback by the thalamus. Moreover, a higher tonic dopamine level promotes the direct pathway with respect to the indirect pathway: this is particularly noticeable in the activity of the Go and NoGo neurons of the winner, being the activity of the first higher and that of the second lower than normal. In case of low tonic dopamine level, the opposite effects are evident, promoting the indirect with respect to the direct pathway. Different levels of tonic dopamine exert differential effect also on the tonic activity of the cholinergic unit ChI: a lower dopamine level increases its activation, whereas higher dopamine levels tend to inhibit it.

In the following simulation, the stimulus S = [0.3  0.3  0.85  0.3] is presented as input to the network, assuming 3 different conditions characterized by a different value of tonic dopamine: the normal value (0.45, blue solid line), a high value (0.55, black dashed line), and a low value (0.35, red dotted line). The simulation is performed with the basal values of all other parameters and synapses (no training was performed before). The winning action is therefore the prepotent one, that is, the third, characterized by the greater excitation for the cortical neurons. Hence, only neuronal activity in the third action channel is displayed for briefness (Figure 9).

As can be easily seen, the tonic level of dopamine has effects on each structure of the network. In the cortex C, the higher the tonic dopamine level, the faster the response, caused by a prompter feedback by the thalamus.

One of the most interesting results is about striatal activity: our model straightforwardly translates the physiological knowledge that tonic dopamine level is associated to an overall imbalance in the direct-indirect pathway [29, 30]. Indeed, a higher tonic dopamine level promotes the direct pathway with respect to the indirect pathway [30, 31]: this is particularly noticeable in the activity of the Go and NoGo neurons of the winner, being the activity of the first higher and of the second lower than normal. This could be interpreted as a possible simulation of traditional medicated Parkinson's disease, as it is generally characterized by higher tonic dopamine assumed by levodopa.

The situation portrayed by the low tonic dopamine level exerts the opposite effects instead, promoting the indirect with respect to the direct pathway: again, the clearest examples are the activities in the Go and NoGo neurons of the winner, which are lower and higher than normal, respectively. This condition could also have a clinical interpretation, as it is widely known that one of the main features of Parkinson's disease is tonic dopamine levels lower than normal.

Moreover, different levels of tonic dopamine exert differential effect also on the tonic activity of the cholinergic unit ChI: lower dopamine levels increase its activation, whereas higher dopamine levels tend to inhibit it.

### 4.7. Sensitivity to Stimuli with Different Tonic Dopamine Levels

The previous simulations clearly showed that a low tonic dopamine level may produce longer response times. In this set of simulations we investigate the relationship between different levels of tonic dopamine and the response latency of our network, using stimuli with different magnitude; the aim is to assess whether there is a relationship between the tonic dopamine level, the subjective sensitivity to the stimuli, and the temporal delay of the gated response.

In these simulations the network is presented with a stimulus S = [0.3  0.3  *a* 0.3], with *a* varying in the range 0.31 ÷ 1. Moreover, 4 different levels of tonic dopamine are adopted: very low, 0.35 (red dotted curve); low, 0.4 (green dot-and-dashed line); physiological, 0.45 (blue solid line); high, 0.55 (black dashed line).

**Figure 10:**
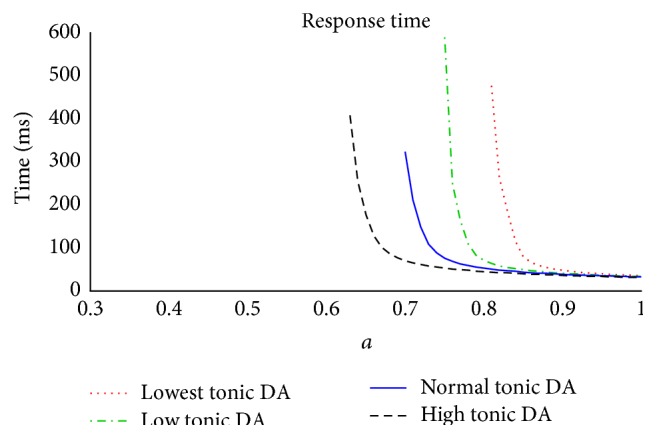
Time delay required to achieve a correct response to a stimulus of a given strength, with very low, low, normal, and high tonic dopamine. The absence of points means that, with the given stimulus and the given dopamine level, no acceptable response was gated by the basal ganglia and so no cortical neuron reached the activation threshold. The simulations show that, in case of stimuli of medium strength (*a* ranging between 0.8 and 0.9), the time required to achieve a valid response crucially depends on the dopamine level: higher levels of dopamine result in faster responses compared with lower levels. Conversely, when the stimulus is high (>0.9), the temporal response is scarcely affected by the dopamine level. Furthermore, in case of low dopamine, the network is able to gate only stimuli of sufficient strength (approximately *a* > 0.8) but it neglects stimuli of lower amplitude.

The results are summarized in Figure 10.

The simulations show that, in case of stimuli of medium strength (*a* ranging between 0.8 and 0.9), the time required to achieve a valid response crucially depends on the dopamine level: higher levels of dopamine result in faster responses compared with lower levels. Conversely, when the stimulus is high (>0.9), the temporal response is scarcely affected by the dopamine level.

Furthermore, in case of low dopamine, the network is able to gate only stimuli of sufficient strength (approximately *a* > 0.8) but it neglects stimuli of lower amplitude. Indeed, the lower the dopamine level, the higher the magnitude of a stimulus necessary for activating the corresponding response.

This again accounts for particular behavior in Parkinson's disease, induced by either low tonic dopamine levels or, after overmedication, tonic dopamine values higher than normal. In the first case, the model predicts that the subject can neglect important responses if the stimuli are not high enough. In the second, hypersensitivity to the stimuli may occur.

## 5. Discussion

The aim of the present work was to develop a new simple model of action selection in the basal ganglia (BG), which could represent a good compromise between completeness and simplicity. Hence, we incorporated the main routes that operate in the BG circuitry and the basic aspects of learning mechanisms involved, still maintaining a simplified description of neural units and neural dynamics.

Actually, many different models of the BG have been developed in the past years, with a great increment in the last decade. As clearly pointed out by Cohen and Frank [12] and Helie et al. [13], biologically inspired models can approximately be subdivided into 2 major classes. On one hand we have anatomically and biophysically detailed models, which include an accurate description of biophysical processes within neurons and synapses (for instance ionic channels); on the other hand, less detailed models try to describe neuron dynamics and synapse learning with more simple and compact equations, still remaining constrained by the neurobiological architecture. The strength of the last class of models is that they can simulate behavioral aspects and may contribute to understand the nature of the computation performed by entire brain regions by relating individual mechanisms with important cognitive neuroscience problems, such as attention, decision, and learning.

The present model definitively belongs to the second class. We tried to include the main mechanisms and neural pathways that participate in the action selection process in the BG, providing a simplified description of neurons and of their reciprocal connections, without the introduction of unnecessary degrees of freedom, in order to mimic the emergent properties of the overall circuitry. In other words, we followed a parsimonious approach, which aspires to realize an efficient tool to understand the functional organization of the system and its behavior in a variety of physiopathological conditions.

Of course, due to the number of good models already present in the literature, it is worthwhile to discuss the innovative aspects of the present study and to point out in which parts it resembles or differs from the existing ones.

In the subsequent analysis, we will focus especially on models for action selection. Only when useful, other models with different aims (like models oriented to study motor functions or working memory) will be mentioned.

Most models of BG developed in past years do not incorporate all the 3 major pathways (direct, indirect, and hyperdirect). For instance, the indirect pathway is not modeled in several important models, such as those by Ashby et al. [32], Moustafa and Gluck [33], Ashby and Crossley [17], or Schroll et al. [34].

Recently, however, several neural cognitive models started including all the 3 main circuitry components, as the present one. In the following section we will refer to these models above all.

Certainly the present model exhibits many similarities and is in debt with various ideas presented in recent papers by Frank and coauthors (Frank [18]; Cavanagh et al. [35]; Wiecki and Frank [36]). In their last models the authors include all the 3 pathways and incorporate the idea (see also Nambu et al. [37]) that the hyperdirect pathway realizes a diffuse inhibition to brake any decision during conditions of high conflict in the cortex. Furthermore, their models make use of Hebb mechanisms in the striatum, based on phasic modulation of the dopamine level.

There are, however, 2 main differences in the present work compared with these similar contributions, which justify our study.

1. First, we included a role for cholinergic neurons in learning, suggesting also that a cholinergic contribution is essential to train synapses in the striatum, especially during punishment. Indeed, Cohen and Frank [12] explicitly discuss the problem of synapse learning during punishment: the question is whether a dip of dopamine can be sufficient for learning, due to the moderate dopamine basal concentration level. These authors support the idea that the* duration* of the dopamine dip is the key element during punishment. Conversely, we propose a different explanation; that is, the dip of dopamine can disinhibit the cholinergic pathway, which, in turn, significantly modulates the activity of the striatal neurons, thus favoring the NoGo (striatopallidal) neurons versus the Go (striatonigral) ones.
2. Second, Frank models use a different mathematical formalism (based on the LEABRA framework), which is in the middle between the use of biophysically detailed neurons and more abstract connectionist neurons. Mathematical equations are described in multiple publications, making it difficult for the reader to synthetize all equations and parameter numerical values as well as their computational implementation. Conversely, the present work reports all equations and parameters in a single paper, thus allowing a straightforward implementation by any user with a basic knowledge on numerical methods for solving differential equations (furthermore, a Matlab version of the model may be available from the authors on request).

Going through the comparison with other authors, Stocco et al. [16] describe a model which mirrors the most important features of the BG anatomy and whose structure is quite similar to the present one. As ours, their model uses simple computational units in the range [0  1]. However, it exhibits important differences as well. First, as in Ashby et al. [32], the model by Stocco simulates the role of dopamine by adding a third term to the Hebb rule. Furthermore, this model does not allow excitation in the prefrontal cortex to be actively maintained due to the absence of recurrent excitatory connections (hence, in their model the cortex cannot be used as a working memory). This is an important difference compared with the present model, with great impact in model dynamics. Indeed, in our model the main role of the BG is to select the best action by consenting activation of the thalamus and hence by triggering a positive feedback between the thalamus and the cortex. For instance, in case of conflicting actions, the role of the hyperdirect pathway is to inhibit all these positive loops, thus avoiding the simultaneous selection of conflicting actions.

A recent model by Ashby and Crossley [17] focuses on the role of cholinergic interneurons, but with fundamental differences. First, the mathematical approach they use belongs to the first class of computational models here exposed (i.e., detailed mathematical description with conductance values); second, the interpretation that the authors give to the role of cholinergic interneurons is different from ours, since they assume these interneurons work only as a “switch,” able to allow the BG to recognize when learning should occur and when not. Hence, their description resembles the “eligibility trace” modeling adopted by others and discussed below.

The model by Chersi et al. [38] also incorporates 3 pathways, with architecture similar to the present one. However, it belongs to the class of more physiologically oriented models, since neurons are described as leaky integrate and fire elements, and a larger number of neurons are adopted (the overall model includes a total of 14600 neurons). More particularly, its learning rule is a combination of 2 components: a spike-timing-dependent plasticity and an eligibility trace. This synaptic plasticity is applied to a greater combination of synapses, including those entering the STN layers and the motor layers (which are not updated in our model). Hence, their model is more realistic than the present at the individual neuronal level, but less parsimonious when it is used with a behavioral purpose.

More similar to the present is the model by Schroll et al. [39], which is also based on 3 pathways and rate neurons, including a learning mechanism based on dopamine. In particular, the model includes plasticity of lateral inhibitory synapses within the Gpi (not present in our model) and of corticothalamic synapses using a two-factor Hebb rule; the other synapses, however, including those converging to the striatum, are learned with a three-factor Hebb rule (i.e., making use of presynaptic, postsynaptic, and dopamine terms). With this model, the authors were able to study the effect of dopamine loss on synaptic plasticity, stressing its role in parkinsonian symptoms.

An important point is that most aforementioned models ([16, 38, 39], but see also Guthrie et al. [40] and Moustafa and Gluck [33]) use a three-factor Hebb rule to train synapses entering striatal neurons. This rule includes the product of 3 terms: a presynaptic, a postsynaptic, and a third multiplicative term which is based on phasic dopamine. An exception is provided by the more recent models by Frank, which do not use a three-factor rule. However, this author uses a more complex rule than the present one, adopting a combination of the Oja rule (i.e., an Hebb rule with a forgetting factor) and an error-driven rule similar to that commonly adopted in Boltzmann machines (for more details, see Frank [18]).

Worth noting is that, in the three-factor rule, the tonic dopamine plays a different role compared with phasic dopamine, acting on the input to neurons and thus setting their working point; that is, tonic and phasic dopamine are conceptually different. Conversely, the Hebb equations for synapse learning in our model do not require a third term but simply use the classic two-factors Hebb rule: dopamine works just on the inputs to neurons (both in the striatum and in the cholinergic interneurons); phasic dopamine differs from tonic dopamine only in its transient nature triggered by reward or punishment events. The basic idea is that, in the absence of phasic dopamine changes, the winner neuron in the striatum works close to the threshold of the postsynaptic term in the Hebb rule. Consequently, no synaptic changes (or just negligible synaptic changes) occur. During reward or punishment, phasic dopamine modifies the activity of striatal neurons, moving the activity of one neuron toward the upper saturation (causing a synapse potentiation) and the activity of other neurons toward inhibition (causing synapse depression). In this regard, it is noticeable that we also included a contrast enhancement mechanism of dopamine in the striatonigral neurons (see also Frank [31]) so that dopamine excites Go neurons with high basal activity but further depresses neurons with poor basal activity. These equations too (equation (8)) are original compared with previous models.

Finally, we wish to stress that the choice of a two-factor rule versus a three-factor rule is not dictated by the need to better reproduce synaptic plasticity but rather to its greater physiological reliability. In our opinion, the dopamine signal used in the three-factor rule is not physiologically well-founded: a simple Hebb rule is more physiologically reliable.

From the previous excursus, we can conclude that the present work introduces 2 main aspects, which are of value, compared with the previous modeling literature:

1. the inclusion of cholinergic mechanisms, especially in the learning phase;
2. the use of a physiological “two-factor” Hebb rule that does not postulate any “eligibility trace.”

Since the cholinergic role in learning is an important new assumption of the present model, it deserves a few further comments. Despite being still partially hypothetical, this mechanism finds some support in the neurophysiological literature. Cholinergic interneurons are tonically active [41] and in primates exhibit a bursting pause during reward [42]. The pause of cholinergic interneurons activity to conditioned stimuli (i.e., a reward) is thought to reflect a linkage with the activity of dopaminergic neurons, as lesioning dopaminergic neurons abolishes both the pause and learning [2, 42]. This idea has been further strengthened by other recent findings, showing that the activity of dopaminergic neurons in primates perfectly mirrors the pause in interneuron activity [43]. Results by Wang et al. [25] also suggest that dopaminergic control of long term depression at striatal synapses is not direct but mediated by cholinergic interneurons. A possible cooperative role of dopamine and acetylcholine in the induction of synaptic plasticity in the striatum has also been stressed for a long time by Calabresi et al. [26]. The present model further hypothesizes that cholinergic interneurons have a different effect on Go and NoGo neurons. Although we are not aware of a direct proof for this specific hypothesis, plausible biochemical mechanisms could justify our assumptions: for instance, it may be sustainable on the basis of both the different role and concentration exerted by muscarinic M1, M2, and M4 receptors in the striatum Go and NoGo [44]. What is known for sure is that cholinergic interneurons play a fundamental role, together with dopamine, in phasic signaling: physiological findings show that this mechanism could be explained by a combined Ca^2+^ and muscarinic M1 effect [24]: this definitely proves that additional inhibition is provided to Go neurons during punishment. In our model we hypothesize that a dual effect is exerted on NoGo neurons, both in reward and punishment.

Several simulations have been performed with the model to illustrate that, with basal parameter values, it behaves as expected. Some of these require further comments.

As previously pointed out by Frank in one of his works [18], simulations in conditions of strong cortical conflict underline the pivotal role played by the hyperdirect pathway through the STN. Nambu et al. [45] proposed that a signal conveyed through the hyperdirect pathway first inhibits large areas of the thalamus. Although our description resembles the one used in Frank [18], we claim that the present simulations are instructive to understand how the mechanism actually works. Results clearly demonstrate that, without the contribution of this hyperdirect signal, the presence of a strong conflict within the cortex could induce the simultaneous excitation of different action channels. In order to avoid this undesired effect (resulting in contradictory actions) a brake excluding the thalamus is needed, to eliminate the strong excitatory self-loop in the cortical winner-takes-all mechanism. This is the same as to remove any positive self-loop in the WTA network during the initial phase of conflict, restoring it again only when the inhibitory intracortical competition has solved (or reduced) the conflict [46].

Other interesting simulations concern the way the model can modify its action selection strategy, depending on the previous history of rewards and punishments in normal conditions. In this regard, the most important synapses in the model are those from the cortex to striatal neurons: we demonstrated that the simple Hebb rule proposed, together with a postsynaptic threshold close to the average neuronal activity, can train these synapses quite well to reach the desired aim. In addition, the synapses form the sensory cortex to the Go neurons are well trained. Conversely, the synapses from the sensory portion of the cortex (S) to the NoGo striatum play a less important role: they are modulated only at the beginning of learning a new action.

Since the introduction of cholinergic mechanisms is a new important feature of the present model, in order to understand the role of cholinergic interneurons in synaptic plasticity we performed a sensitivity analysis assuming a lesioned cholinergic system. As expected, these further simulations strongly prove and emphasize the role of the cholinergic interneurons during training. In fact, in their absence, an insufficient depotentiation of synapses entering the punished Go neurons and a slower potentiation of synapses entering the rewarded neuron can easily be observed. As a consequence, punished actions remain active and compete with the rewarded actions.

It is worth noting that when performing our simulations we assumed that the external stimulus does not merely consists in the activation of a single input, but that more inputs can be simultaneously excited. In other words, here the stimulus vector S represents a* context*, within which the proper action must be selected, not just a single pointy input (in fact, in the simulations shown in Figure 7(c), both the third and fourth components of S are active). As is well known, a problem may arise if nonorthogonal stimuli are associated during training with different responses, since an interference may occur between previously learned stimulus-responses associations [47]. We did not test this problem in the present work, because we just wanted to analyze single stimulus-response learning. The problem may be investigated in future model applications. In case, a possible interference might be solved, as usually done in neural network models, assuming a preprocessing net which orthogonalizes the stimulus vectors, in order to reduce correlation and using a larger number of input neurons.

Furthermore, we tested the role of tonic dopamine in the model. This is extremely important to simulate pathological conditions, like those occurring in Parkinson's disease (PD) subjects experiencing dopamine depletion or dopamine hypermedication. Results clearly show that a reduction of the tonic dopamine level causes various behavioral deficits: first, a stronger stimulus may be required to elicit the same behavioral response; second, in case of sufficient stimulus, triggering the same response requires a longer time. Both these results agree with clinical findings, showing that tonic dopamine is important to speed the reaction times [48, 49].

Since the model uses just a single dopamine term, which acts on striatal and cholinergic neuron inputs, a similar effect of dopamine depletion is expected in the learning phase too. This characteristic will be tested in future model applications, also comparing model predictions with behavioral data. Among the different behavioral tests that may be simulated with the model and are often used in the clinical practice on PD patients, we can mention the finger tapping test [50] and the Wisconsin card sorting test [51]: indeed the ability to perform these tests is often compromised in PD and is significantly correlated with the pharmacokinetic profile of oral Levodopa [52, 53].

There are many other behavioral results, concerning subjects with PD, which can be explored with our model in future works. For instance, it would be interesting to compare the differential sensitivity to rewards and punishments between medicated and unmedicated PD subjects, and the model could also be used to test the effect of anticholinergic medications on PD subjects a well. These analyses may further confirm model assumptions on the learning role of dopamine and acetylcholine or may lead to possible useful corrections.

Finally, we wish to underline a few simplifications of the present model, which may become target of future improvement. First, we did not adopt a temporal differential learning rule (Sutton [54], Suri [55]): the model just uses a delayed associative learning rule, based on the values at the end of the trial to modify the synapses. Furthermore, the punishment and reward signal are treated as external inputs. There is large consensus today that phasic dopamine changes are especially caused by “unexpected rewards” and by “unexpected absences of reward” [2, 20, 54, 56–59]. This means, for instance, that we should use a temporal modulation of rewards and punishments during training, thus progressively reducing the phasic dopamine peaks when a strategy has been successfully learned; that is, the reward becomes well-expected. A more sophisticated future model strategy may incorporate a temporal rule and maybe include a division between a critic and an actor, to realize more complex learning strategies. Indeed, only the actor is implemented in the present model version. Furthermore, as discussed above, the assumption that acetylcholine has different effects on Go and NoGo cells is still partially hypothetical. Finally, the model assumes the convergence of the sensory and motor (cortical) inputs to the same cells of the striatum: this hypothesis is still not fully supported by anatomical data.

Lastly, the model does not incorporate some assumptions. For instance, the classical view that the BG are functionally organized into the previously mentioned 3 main pathways is acknowledged, as the majority of computational models assume. This vision has been recently questioned providing proofs and examples of other plausible functional interpretations [60]. As minor points, the model neglects feedback projections from the thalamus to the striatum and from the Gpe to the striatum. This simplification, however, is common to most models (see, for instance, Stocco et al. [16]). Some authors trained also cortico-cortical synapses (see Cohen and Frank [12]): as discussed by these authors, this may allow habits often chosen in the past to be incorporated directly within the cortex, without the need for any facilitation by the BG.

## Figures and Tables

**Table 1:** Parameter values of the model in the default state and initial values of state variables.

Name	Value
*τ*/*τ*_*L*_	10 [ms]/50 [ms]
*a*	4
*u*_0_	1
*ϑ*_G_	0.3
*I* ^E^	1
*I* ^I^	3
*I* ^H^	1.25
*α*	1
*β*	−1
*γ*	−1
*σ*	0.1
*ϑ*_PRE_	0.5
*ϑ*_POST_	0.5

**Table 2:** Synaptic values of the model (pretraining).

Name	Projection	Type	Values
*L*	Inhibition	Extradiagonal matrix	liji≠j=-1.2
*W* ^CS^	Excitation	Full matrix	*w*_*ii*_^CS^ = 1.1; wiji≠jCS=0.2
*W* ^CT^	Excitation	Diagonal matrix	*w*_*ii*_^CT^ = 4
*W* ^GC^	Excitation	Diagonal matrix	*w*_*ii*_^GC^ = 0.48
*W* ^GS^	Excitation	Full matrix	*w*_*ii*_^GS^ = 0.9; wiji≠jGS=0
*W* ^NC^	Excitation	Diagonal matrix	*w*_*ii*_^NC^ = 1.08
*W* ^NS^	Excitation	Full matrix	*w*_*ii*_^NS^ = 0.1; wiji≠jNS=0
*W* ^EN^	Inhibition	Diagonal matrix	*w*_*ii*_^EN^ = −2.2
*W* ^IE^	Inhibition	Diagonal matrix	*w*_ii_^IE^ = −3
*W* ^IG^	Inhibition	Diagonal matrix	*w*_*ii*_^IG^ = −12
*W* ^TC^	Excitation	Diagonal matrix	*w*_*ii*_^TC^ = 3
*W* ^TI^	Inhibition	Diagonal matrix	*w*_*ii*_^TI^ = −3
*w* ^ESTN^	Excitation	Scalar	*w* ^ESTN^ = 1
*w* ^ISTN^	Excitation	Scalar	*w* ^ISTN^ = 14
*k* ^E^	Excitation	Scalar	*k* ^E^ = 7
*W* ^STNE^	Inhibition	Row vector	*w*_*i*_^STNE^ = −1
*w* ^GH^	Inhibition	Scalar	*w* ^GH^ = −1
*w* ^NH^	Excitation	Scalar	*w* ^NH^ = 1
